# Optimized protocol for direct extraction of SARS-CoV-2 RNA from raw wastewater samples (ANRS 0160)

**DOI:** 10.1016/j.mex.2025.103323

**Published:** 2025-04-18

**Authors:** Ahlam Chaqroun, Ghina El Soufi, Zuzana Gerber, Julie Loutreul, Nicolas Cluzel, Julie Challant, Damien Delafoy, Florian Sandron, Léo Di Jorio, Stéphanie Raffestin, Vincent Maréchal, Christophe Gantzer, Robert Olaso, Jean-François Deleuze, Olivier Rohr, Nicolas Boudaud, Clémentine Wallet, Isabelle Bertrand

**Affiliations:** a Université de Lorraine, CNRS, LCPME, F-54000 Nancy, France; b Université de Strasbourg, UPR CNRS 9002 ARN, F-67300 Schiltigheim, France; c Université de Strasbourg, IUT Louis Pasteur, F-67300 Schiltigheim, France; d CEA, Centre National de Recherche en Génomique Humaine, Université Paris-Saclay, F-91057 Evry, France; e ACTALIA, F-50000 Saint Lô, France; f Maison des Modélisations Ingénieries et Technologies (SUMMIT), Sorbonne Université, F-75005 Paris, France; g Institut Pasteur de la Guyane, French Guiana, F-97300 Cayenne, France; h INSERM, Centre de Recherche Saint-Antoine, Sorbonne Université, F-75012 Paris, France; i OBEPINE consortium, Paris, France

**Keywords:** PCR inhibitors, RNA extraction, SARS-CoV-2, Silica beads, Silica columns, Wastewater, NS2

## Abstract

This study aimed to evaluate whether a nucleic acid extraction protocol specifically designed for raw wastewater (WW) provides a measurable advantage over protocols not originally intended for WW matrices. Three laboratories have independently compared two RNA extraction protocols, using paired WW samples. A common WW-designed protocol (Z), based on silica columns was tested across all labs. As comparators, three non-WW designed protocols were used: NS1 and NS2 both silica beads-based, with NS2 including an additional phenol-chloroform step; and SB a homemade protocol using also silica beads but differing in its formulation. As different samples were used across labs, direct statistical comparison was difficult; instead, paired comparisons within each lab were used to rank SARS-CoV-2 RNA detection and RT-qPCR inhibitor removal. NS1 yielded significantly higher SARS-CoV-2 RNA concentrations than Z (Logrank test, *p* ≤ 0.001), though with RT-qPCR inhibition in one sample. NS2 also showed higher SARS-CoV-2 RNA detection than Z (Wilcoxon test, *p* < 0.0001), with both protocols showing complete inhibitor removal. SB performed worse than Z for SARS-CoV-2 RNA detection (Logrank test, *p* ≤ 0.05), and showed inhibition in one sample. NS2 was the most effective option for both RNA detection and inhibitor removal.

## Graphical abstract

**Figure:**
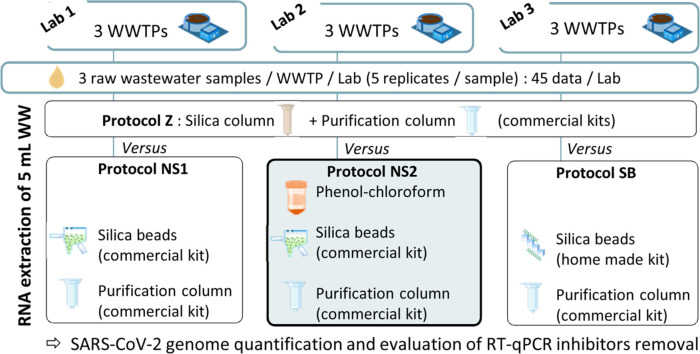

Specifications table

**Table:** 

Subject area:	Immunology and Microbiology
More specific subject area:	Environmental microbiology
Name of your protocol:	NS2
Reagents/tools:	Separation tube (50 mL conical tube): Each separation tube contained 4 g of a mixture (90:10 w/w) made of silicon grease (Molykote™ High Vacuum Grease, Cole Parmer, Ref. 79751-30) and silicon dioxide (Sigma-Aldrich, Ref. 1075361000). Phenol-chloroform-isoamyl alcohol (25:24:1 pH 7.8–8.2, Sigma Aldrich, Ref. 177617). Centrifugation during phenol-chloroform treatment was performed with a swinging bucket rotor centrifuge (Heraeus Multifuge X3 FR) NucliSENS^Ⓡ^ miniMAG™, bioMérieux NucliSENS^Ⓡ^ easyMAG^Ⓡ^ automated system, bioMérieux NucliSENS^Ⓡ^ kit, bioMérieux: NucliSENS^Ⓡ^ easyMAG^Ⓡ^ Lysis Buffer (1000 mL), Ref. 280134 NucliSENS^Ⓡ^ easyMAG^Ⓡ^ Magnetic silica, Ref. 280133 NucliSENS^Ⓡ^ easyMAG^Ⓡ^ Extraction Buffer 1, Ref. 280130 NucliSENS^Ⓡ^ easyMAG^Ⓡ^ Extraction Buffer 2, Ref 280131 NucliSENS^Ⓡ^ easyMAG^Ⓡ^ Extraction Buffer 3, Ref. 280132 Magnetic rack for 1.5-mL tubes: Promega MagneSphere 12-Tube Magnetic Separation Stand, Ref. Z5342 Magnetic rack for 50-mL tubes: PolyATtract^Ⓡ^ System 1000 Magnetic Sep, Ref. Z5410 OneStep PCR Inhibitor Removal kit, ZymoResearch, Ref. D6030
Experimental design:	Two viral RNA extraction protocols were compared using raw WW samples: a WW designed protocol against non-WW designed protocols (NS1, NS2 or SB). The protocol NS2 was the only effective option for both RNA detection and inhibitor removal, like the Z protocol. This protocol combines a neutral phenol chloroform treatment followed by RNA capture and purification using magnetic silica beads, and ends with a final purification step on resin column.
Trial registration:	Not applicable
Ethics:	Not applicable
Value of the Protocol:	• Highly efficient recovery of viral RNA from raw WW samples • Complete removal of RT-qPCR inhibitors • Can be easily combined with virus concentration methods such as ultrafiltration

## Background

The extraction of viral RNA from raw wastewater (WW) samples is crucial for viral surveillance and research. These environmental samples contain a complex mix of organic and inorganic materials that can interfere with RNA extraction, complicating the detection of viruses present at low concentrations. Selecting an appropriate extraction protocol is essential to ensure high yield and purity of viral RNA. These parameters directly affect the sensitivity and reliability of downstream analyses such as PCR based methods, and sequencing analysis. In wastewater-based epidemiology, the most commonly used RNA extraction methods include column-based kits (e.g., QIAamp Viral RNA Mini Kit, RNeasy Mini Kit—Qiagen), organic extraction methods (e.g., TRIzol reagent—Invitrogen), and magnetic bead-based protocols (e.g., MagMAX Viral/Pathogen Nucleic Acid Isolation Kit—Thermo Fisher Scientific; NucleoMag RNA Kit—Macherey-Nagel), each offering specific advantages and limitations depending on the complexity of the matrix, resource availability, and the downstream application [2,3]. Organic-based extraction methods, such as TRIzol or home-made protocols using guanidinium thiocyanate, phenol, and chloroform, are capable of extracting RNA in high purity and quantity [[4], [5], [6], [7]]. However, they involve the use of hazardous chemicals, require careful handling, and can be time-consuming and labor-intensive, particularly in their non-commercial forms. Silica column-based extraction is widely favored for its simplicity, speed, and effectiveness in isolating high-purity RNA [6]. It involves binding nucleic acids to silica membranes in the presence of chaotropic salts, followed by wash steps to remove contaminants. A potential drawback of this method is that processing overly concentrated or large-volume samples, which can clog the column and reduce both yield and RNA quality [6]. Magnetic bead-based extraction uses silica-coated magnetic particles and is well-suited for high-throughput applications and automation. It delivers high RNA purity and recovery, though the main limitation is the higher cost associated with automated systems [7,8]. The aim of this study was to compare a WW-designed RNA extraction protocol based on silica columns with non-WW-designed protocols using silica beads, across three different laboratories with different expertise with each of the protocols. The evaluation focused on SARS-CoV-2 RNA detection efficiency and the removal of RT-qPCR inhibitors using a 5 mL volume of WW.

## Description of protocol

**Fig. 1:**
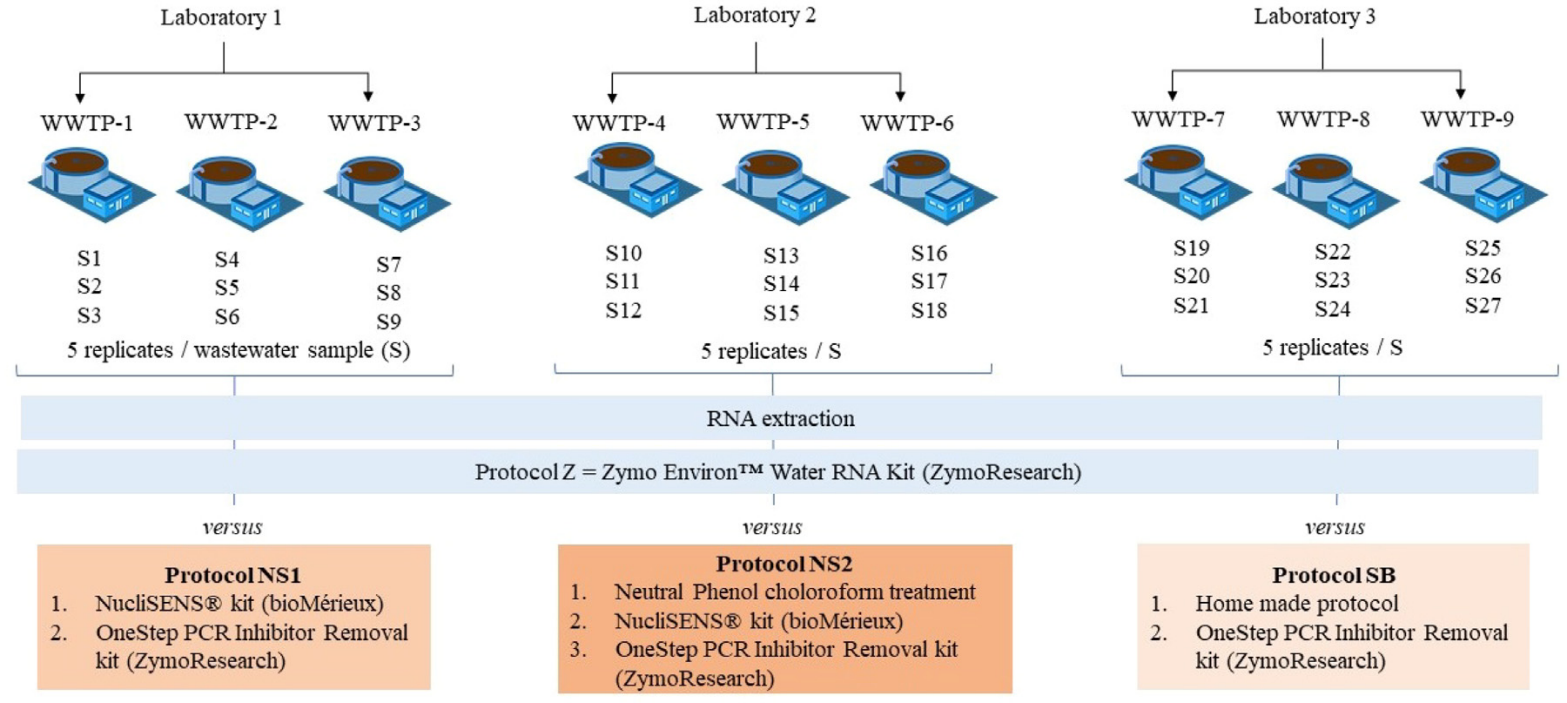
Comparison of WW designed protocol (Z) against non-WW designed protocols (NS1, NS2 or SB) on WW samples (5 mL). Laboratory 1 compared the protocol Z (silica columns extraction protocol) with the protocol NS1 (NucliSENS extraction protocol) on 9 influent samples (S1 to S9) obtained from 3 different WWTPs. Laboratory 2 compared the Z protocol with the NS2 protocol on another set of 9 influent samples (S10 to S18) from 3 WWTPs. Laboratory 3 compared the Z protocol with the SB protocol (homemade silica beads extraction protocol) on a third set of 9 influent samples (S19 to S27) from 3 WWTPs. For each sample, 5 replicates were performed for RNA extraction protocol. WWTP: Wastewater treatment plant; WW: Wastewater; S: WW sample; FRNAPH GGII: F-specific RNA phages genogroup II.

Three laboratories compared the WW designed protocol (Z) against non-WW designed protocols, resulting in three independent comparison sets, each evaluating paired WW samples collected and processed by the participating laboratory. Laboratory 1 compared Z with NS1, Laboratory 2 with NS2, and Laboratory 3 with SB. The comparison was conducted on WW samples naturally contaminated with SARS-CoV-2 and F-specific RNA phages (FRNAPH) with the experimental design outlined in Fig. 1. All wastewater samples were collected from urban WWTPs over 24-hour periods using automated samplers, ensuring they were 24-hour composite samples representative of daily influent flow. Each participating laboratory collected and processed its own samples locally. Upon collection, samples were stored at 4 °C and processed within a maximum of 48 h. Aliquots of 5 mL were prepared from raw WW for direct RNA extraction. These procedures were applied consistently across all laboratories to minimize variability and ensure comparability of results. WW sample location characteristics are outlined in Table 1.

**Table 1:** Influent samples and related WWTP.

WWTP	Location (region)	Capacity (inhabitants equivalent)	Sampling period	Number of influent samples
WWTP-1	Grand Est	400,000	March-May 2022	9
WWTP-2	Nouvelle Aquitaine	150,000	March-May 2022	9
WWTP-3	Nouvelle Aquitaine	135,000	March-May 2022	9
WWTP-4	Normandie	332,000	April-June 2022	9
WWTP-5	Auvergne-Rhône-Alpes	50,000	April-June 2022	9
WWTP-6	Occitanie	160,000	April-June 2022	9
WWTP-7	Grand Est	21,000	May-June 2022	9
WWTP-8	Grand Est	30,000	May-June 2022	9
WWTP-9	Grand Est	20,000	May-June 2022	9

**ABBREVIATIONS LIST**

• **BSL-2** – Biosafety Level 2
• **Cq** – Quantification Cycle
• **CV** – Coefficient of Variation
• **FRNAPH GGII** – F-Specific RNA Phages Genogroup GGII
• **GC** – Genome Copies
• **ISO** – International Organization for Standardization
• **LOD** – Limit of Detection
• **LOQ** – Limit of Quantification
• **NPC** – Neutral Phenol-Chloroform
• **NS1** – NucliSENS^Ⓡ^ Protocol 1
• **NS2** – NucliSENS^Ⓡ^ Protocol 2
• **PPE** – Personal Protective Equipment
• **qPCR** – Quantitative Polymerase Chain Reaction
• **RNA** – Ribonucleic Acid
• **RT-PCR** – Reverse Transcription Polymerase Chain Reaction
• **RT-qPCR** – Reverse Transcription Quantitative Polymerase Chain Reaction
• **SB** – Silica Beads home-made protocol
• **WB1** – Wash Buffer 1
• **WB2** – Wash Buffer 2
• **WB3** – Wash Buffer 3
• **WW** – Wastewater

**WARNINGS AND PRECAUTIONS:**

• WW samples may contain hazardous pathogens. Handle all samples as biosafety level 2 (BSL-2) materials. Always wear personal protective equipment (PPE), including gloves, lab coat, and safety goggles.
• Phenol-chloroform-isoamyl alcohol is highly toxic, caustic, and volatile. Work in a chemical fume hood and wear appropriate PPE. Avoid skin and eye contact, in case of exposure, rinse immediately with copious amounts of water.

**NB:**

• all tested protocols followed the Manufacturer's Protocol
• all tested protocols used the same RNA elution volume of 50 µL

**RNA EXTRACTION PROTOCOLS**

**1.** NS2 protocol using NuclisENS^Ⓡ^ kit

*Sample Preparation and lysis*

• Add 10 mL of NucliSENS^Ⓡ^ lysis buffer to 5 mL of WW sample.
• Homogenize for 1 min using a vortex at high-speed settings (7–8).
• Incubate the mixture at room temperature for 10 min.
• The resulting mixture is referred to as the lysate.

*Neutral Phenol-Chloroform(NPC)Treatment (theonly modification to Manufacturer's Protocol)*

• Prepare a separation tube in a 50 mL conical tube containing: 4 g of a mixture of high-vacuum silicon grease and silicon dioxide in a 90:10 w/w ratio.
• Transfer 15 mL of lysate into the separation tube.
• Add 15 mL of phenol-chloroform-isoamyl alcohol (25:24:1 pH 7.8–8.2, Sigma Aldrich, Ref. 77617).
• Shake vigorously by hand for 1 min.
• Centrifuge at 3500 × *g* for 5 min.
• Transfer 15 mL of the aqueous phase into new 50 mL conical tube.

*RNA Binding toMagneticSilica Beads*

• Add 70 µL of magnetic silica beads (from the NucliSENS^Ⓡ^ kit) to the aqueous phase.
• Homogenize the mixture by vortex for 1 min at high-speed settings (7–8).
• Incubate at room temperature for 10 min.
• Place the tube with the mixture in a magnetic rack and discard the fluid.

*RNA Extraction Options*

The RNA extraction process can proceed via NucliSENS^Ⓡ^ miniMAG^TM^, NucliSENS^Ⓡ^ easyMAG^Ⓡ^ automated system, or manual magnetic racks.

**Option 1:** Using NucliSENS^Ⓡ^ miniMAG^TM^

• Resuspend the silica magnetic beads with 400 µL of Wash Buffer 1 (WB1).
• Transfer the suspension to a 1.5 mL tube and place it in the NucliSENS^Ⓡ^ miniMAG^TM^.
• Perform two washing steps:
  - Contact time: 30 s per wash.
  - Discard fluid via aspiration after each step.
• Perform two additional washes with 500 µL of Wash Buffer 2 (WB2):
  - Contact time: 30 s per wash.
  - Discard fluid via aspiration after each step.
• Perform a final wash with 500 µL of Wash Buffer 3 (WB3):
  - Contact time: 15 s.
  - Discard fluid via aspiration after each step.
• Remove the 1.5 mL tube from the NucliSENS^Ⓡ^ miniMAG^TM^.
• Resuspend the silica beads in 50 µL of WB3.
• Perform elution at 60 °C for 5 min at 1400 rpm in a Thermomixer comfort (Eppendorf).
• Place the tube in a magnetic rack and collect the 50 µL of eluate.

**Option 2:** Using NucliSENS^Ⓡ^ easyMAG^Ⓡ^ Automated System

• The same washing and elution steps are performed automatically.
• WB3 is only used for elution in the easyMAG^Ⓡ^ system.

**Option 3:** Manual RNA Extraction

• Use magnetic racks adapted to 1.5 mL and 50 mL tubes to manually process the magnetic silica beads through the washing and elution steps.

*RNA Purification (OneStepTM PCR Inhibitor Removal)*

• Prepare Zymo-SpinTM III-HRC columns:
  - Place each column into a collection tube.
  - Add 600 µL of Prep-solution to the column matrix.
  - Centrifuge at 8000 × *g* for 3 min.
• Transfer the prepared column into a clean 1.5 mL tube.
• Load the 50 µL extracted RNA onto the column.
• Centrifuge at 16,000 × *g* for 3 min.
• The eluated RNA is ready for RT-PCR analysis.

**2.** NS1 protocol using NucliSENS^Ⓡ^ Kit

The only difference between NS1 and NS2 is the absence of NPC treatment.

Steps Unique to NS1:

• Instead of NPC treatment, the lysate is centrifuged at 2500 × *g* for 5 min.
• The supernatant (14.5 mL) is transferred to a new 50 mL conical tube.
• 70 µL of magnetic silica beads are added to the supernatant and homogenized via vortex for 1 min.
• All other steps remain identical to NS2.

**3.** Z protocol using Zymo Environ™ Water RNA Kit

*Sample Preparation*

• Mix 350 µL of Water Concentrating Buffer™ with 5 mL of WW sample.
• Vortex the mixture for 1 min.
• Incubate at room temperature for 10 min.

*Centrifugation and Pellet Collection*

• Centrifuge at 3000 × *g* for 15 min.
• Carefully pipette out the supernatant without disturbing the pellet, leaving 250 µL of pellet.

*Lysis and Bead Beating*

• Add 750 µL of DNA/RNA Shield™ to the pellet, mix by vortex for 1 min.
• Transfer the 1 mL mixture into a ZR BashingBead™ Lysis Tube.
• Vortex in a bead beater (Vortex-Genie 2, Scientific Industries) for 1 min at high-speed settings (7–8).
• Centrifuge at 12,000 × *g* for 2 min.

*RNA Binding and Column Transfer*

• Collect 400 µL of supernatant after centrifugation.
• Mix with an equal volume of RNA Binding Buffer.
• Vortex for 30 s.
• Transfer the mixture to a Zymo-Spin™ IIICG Column.
• Centrifuge at 16,000 × *g* for 30 s.

*Ethanol Precipitation*

• Combine the flow-through with an equal volume of absolute ethanol.
• Vortex for 30 s.
• Transfer the mixture to a new Zymo-Spin™ IIICG Column.
• Centrifuge at 16,000 × *g* for 30 s.

*RNA Wash and Elution Preparation*

• Add 400 µL of RNA Prep Buffer to the column.
• Centrifuge at 16,000 × *g* for 30 s.
• Transfer the column to a new RNase-free tube.
• Add 100 µL of DNase/RNase-Free Water to the column matrix.
• Centrifuge at 16,000 × *g* for 30 s.

*Final Purification Using Zymo-Spin™ III-HRC Filter*

• Place a Zymo-Spin™ III-HRC Filter into a new Collection Tube.
• Pre-treat the filter by adding 600 µL of Prep Solution.
• Centrifuge at 8000 × *g* for 3 min.
• Discard the flow-through.
• Transfer the eluted RNA from the previous step into the prepared Zymo-Spin™ III-HRC Filter in an RNase-free tube.
• Centrifuge at 16,000 × *g* for 3 min.

*Second RNA Binding and Column Purification*

• Mix the filtrate with 200 µL of RNA Binding Buffer and 300 µL of absolute ethanol.
• Transfer the mixture to a Zymo-Spin™ IC Column in a Collection Tube.
• Centrifuge at 16,000 × *g* for 30 s.
• Discard the flow-through.

*Washing Steps*

• Add 400 µL of RNA Prep Buffer to the column.
• Centrifuge at 16,000 × *g* for 30 s.
• Wash the column with 700 µL of RNA Wash Buffer.
• Centrifuge at 16,000 × *g* for 30 s.
• Discard the flow-through.
• Wash the column again with 400 µL of RNA Wash Buffer.
• Centrifuge at 16,000 × *g* for 2 min to ensure complete removal of the wash buffer.

*Final Elution*

• Transfer the column carefully into an RNase-free tube.
• Add 50 µL of DNase/RNase-Free Water to the column.
• Centrifuge at 16,000 × *g* for 30 s.
• The eluted RNA is ready for downstream applications such as RT-PCR analysis.

**4.** SB protocol: The protocol SB is based on silica beads and was previously described [9].

**STORAGE OF EXTRACTED RNA:** Following extraction, all RNA samples were stored at −80 °C until they underwent viral genome quantification of SARS-CoV-2 RNA and evaluation of RT-qPCR inhibition by quantifying the genome of F-specific RNA phages belonging to genogroup GGII (FRNAPH GGII).

**SARS-COV-2 RNA QUANTIFICATION**

*Preparation of the RT-qPCR Reaction*

• Use the RNA UltraSens™ One-Step Quantitative RT-PCR kit (Applied Biosystems™).
• Prepare the reaction mixture in a final volume of 20 µL containing: 2 µL of extracted RNA sample; 1 µM of primers targeting the E gene of SARS-CoV-2 [10] and 0.3 µM of the probe.

*RT-qPCR Amplification Conditions*

• Perform amplification using a BioRad CFX96™ thermal cycler with the following cycling parameters:
  - Reverse Transcription Step: 50 °C for 30 min.
  - Initial Denaturation: 95 °C for 5 min.
  - PCR Amplification (45 Cycles): 95 °C for 15 s and 58 °C for 40 s.

*Quantification Using a Standard Curve*

• A known target sequence in this case, the SARS-CoV-2 E gene amplicon cloned into a plasmid vector called pCR2.1 (Invitrogen, 452,640) [11] was used for quantification.
• This plasmid acts as a reference standard because it contains the exact sequence of interest.
• A 10-fold serial dilution of the plasmid is prepared. Each dilution contains a known copy number of the target gene. The diluted plasmid samples are run in RT-qPCR to generate Cq (quantification cycle) values.
• A standard curve is plotted with: The logarithm of the known copy number (x-axis) and the corresponding Cq value (y-axis).
• The curve allows for linear regression analysis, which is then used to calculate the unknown RNA concentration in test samples based on their Cq values.

*Determination of Limit of Detection (LOD) and Limit of Quantification (LOQ)*

• The LOD and LOQ were determined using Synthetic SARS-CoV-2 RNA Control 2 (Twist Bioscience, ref MN908947.3).
• LOD (Limit of Detection) is defined as the lowest RNA target concentration with 95 % detection success [[12], [13], [14]]. It was set at 10 GC/reaction based on 6 replicates.
• LOQ (Limit of Quantification) is defined by the user as there is no standardized coefficient variation (CV) for qPCR [12,14]. It was set at 10 GC/reaction with a CV of 25 %.

**PCR INHIBITOR REMOVAL EVALUATION**

*RT-qPCR Assay for FRNAPH-II*

• The FRNAPH-II genome was quantified using an RT-qPCR assay (VTB4-Fph II set, [15]). These non-enveloped phages are naturally present in wastewater influent at concentrations of 10⁵ to 10⁹ GC/L [16,17].
• The RNA UltraSens™ One-Step Quantitative RT-PCR kit was used under the same cycling conditions as described for SARS-CoV-2.

*Assessing RT-qPCR Inhibition*

• RNA quantification was performed for undiluted extracts and 1/10 diluted extracts.
• Cq (quantification cycle) values were compared between the two extracts.

*Inhibition Estimation Criteria*

• RT-qPCR inhibition was assessed for all extracted RNA samples by comparing the quantification cycle (Cq) values between undiluted and 1:10 diluted extracts.
• The inhibition percentage is based on the difference in Cq (ΔCq) values for the same sample, if the ΔCq:
  - Reaches 2.3, 50 % of samples are inhibited.
  - Reaches 1.3, 75 % of samples are inhibited.
• Acceptable Inhibition Level: according to ISO 15216-1 (2017) (used for norovirus and hepatitis A quantification in food/water matrices), up to 75 % inhibition is acceptable.
• This approach was applied consistently across all protocols and laboratories.

## Protocol validation

The comparison was performed in parallel in three laboratories; each laboratory compared protocol Z designed for environmental samples to one of the three others (SB, NS1 or NS2) (Fig. 1). Three comparative sets were prepared: Z versus NS1 (Laboratory 1), Z versus NS2 (Laboratory 2), and Z versus SB (Laboratory 3). Each laboratory used raw WW samples collected in three WWTPs with three distinct samples per WWTP (*n* = 9 per laboratory) (Table 1). As each sample underwent five technical replicates, the comparative set of each lab contained a total of 45 data and the global dataset contained 135 data. The comparison of the RNA extraction protocols was based on two key factors: the quantification of the SARS-CoV-2 RNA and the removal of RT-qPCR inhibitors by targeting FRNAPH belonging to genogroup II (FRNAPH GGII). The molecular biology analyses were performed as previously described in the original research article [1]. RT-qPCR inhibition was estimated for each sample of extracted RNA by comparing the quantification cycle (Cq) values obtained for the undiluted extract with those of the 1/10 diluted extract. If the difference between the two Cq values (ΔCq) reached 2.3, then the inhibition was 50 %. If this difference reached 1.3, then the inhibition was 75 %. According to ISO 152616-1 (2017) on the genome quantification of noroviruses and hepatitis A virus in food and bottled water matrices, up to 75 % inhibition is acceptable. Statistical analyses were performed using GraphPad - Prism software (version 9.5.0). Each laboratory compared two extraction protocols on paired samples. The Wilcoxon test (*n* = 2 series) was employed to compare viral RNA genome copies and RT-qPCR inhibitor removal for the dataset of each lab. Negative samples were included in the statistical comparison, and the Logrank test for paired samples (*n* = 2 series) was applied in this case. Results with p-values < 0.05 were considered statistically significant, denoted by asterisks (* for *p* ≤ 0.05; ** for *p* ≤ 0.01; *** for *p* ≤ 0.001; **** for *p* ≤ 0.0001); 'ns' indicated non-significant differences.

**Fig. 2:**
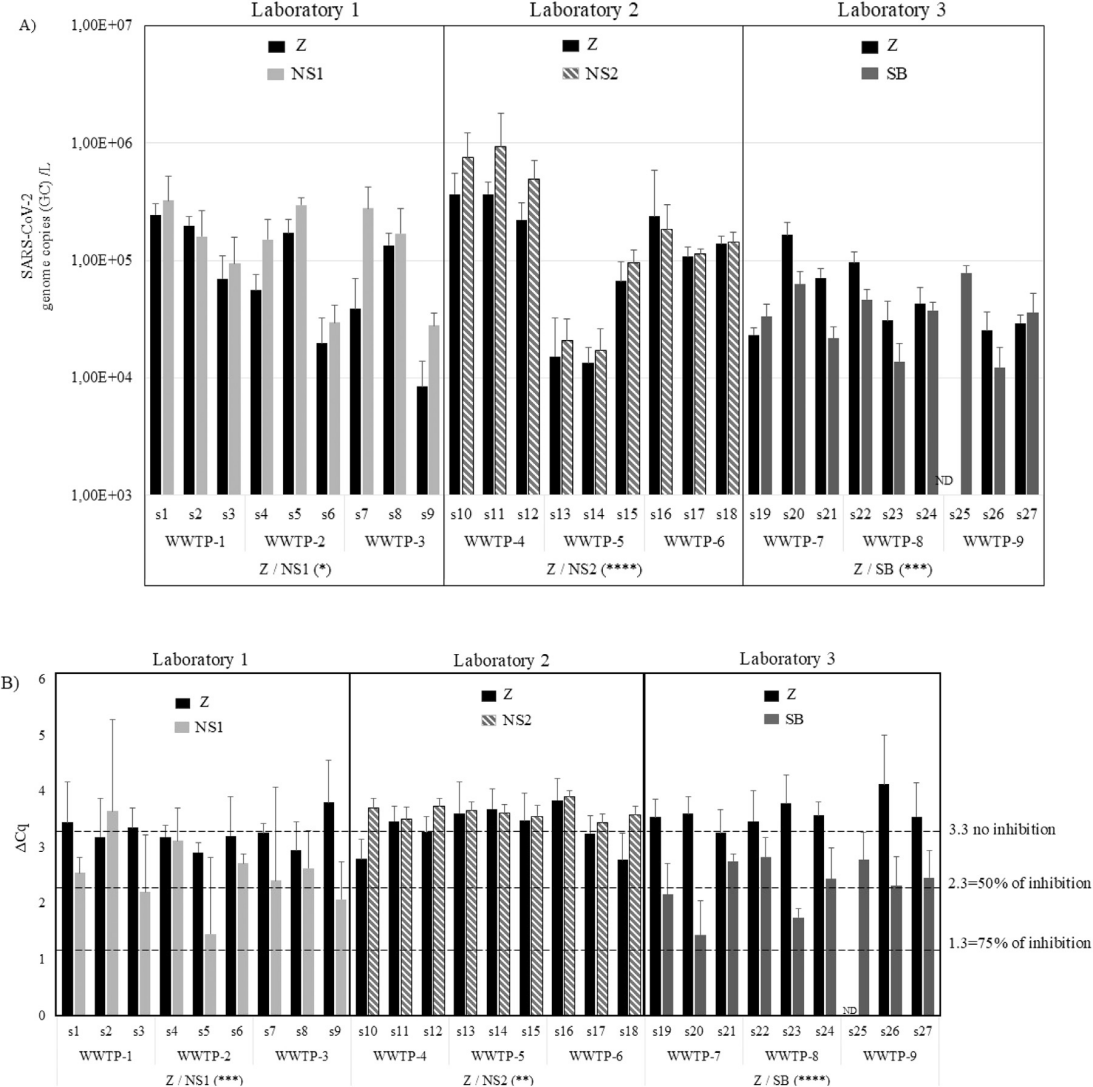
Comparison of WW designed protocol (Z) against non-WW designed protocols (NS1, NS2 or SB) on WW samples (5 mL). The comparison was done two by two, with each laboratory (laboratories 1, 2 and 3) comparing the Zymo protocol to another protocol. A) Comparison of the concentrations of SARS-CoV-2 RNA genome (GC/L). B) Comparison according to ∆Cq value, which is the difference between undiluted extract and the 1/10 diluted extract. s1 to s27 refer to the WW samples analyzed by each laboratory (s1 to s9: laboratory 1; s10 to s18: laboratory 2; s19 to s27: laboratory 3). Each bar indicates the mean value of five technical replicates from the same sample. The standard deviation is also given for each sample. ND = not detected. A statistical Wilcoxon test was used to compare the two protocols used in each laboratory, significant differences are represented by asterisks (*: *p* ≤ 0.05; ***: *p* ≤ 0.001 and ****: *p* ≤ 0.0001).

The genome concentration values of SARS-CoV-2 and the ΔCq values of FRNAPH GGII are represented in Fig. 2A and Fig. 2B, respectively. The data are also available in Table 2. The comparison of the Z and NS1 protocols showed significantly higher concentrations of SARS-CoV-2 RNA using the NS1 protocol (1.6 × 10^4^ to 6.6 × 10^5^ GC/L) than the Z protocol (3.7 × 10^3^ to 3.3 × 10^5^ GC/L) (Logrank test, p value < 0.01). The presence of RT-qPCR inhibitors was detected only for the NS1 protocol, in one sample set (75 % of inhibition in S5 from WWTP-2). In the comparison between the Z and NS2 protocols, NS2 also yielded significantly higher concentrations of SARS-CoV-2 RNA (5.7 × 10^3^ to 2.5 × 10^6^ GC/L) compared to the Z protocol (3.6 × 10^3^ to 8.6 × 10^5^ GC/L) (Wilcoxon test, p value < 0.0001). Additionally, the NS2 protocol removed all RT-qPCR inhibitors, as did the Z protocol. In a previous study [18], an improvement of the removal of RT-qPCR inhibitors was also observed by adding the phenol-chloroform treatment to the NucliSENS^Ⓡ^ kit. The comparison between the Z and SB protocols showed lower concentrations of SARS-CoV-2 RNA genome for the SB protocol (5.3 × 10^3^ to 9.2 × 10^4^ GC/L) compared to the Z protocol (9.3 × 10^3^ to 2.2 × 10^5^ GC/L) (Logrank test, p value = 0.03311987). Additionally, the SB protocol exhibited about 75 % inhibition in one sample set (in S20 from WWTP-7).

**Table 2:** Comparing RNA extraction protocols from small influent Volume (5 mL).

Lab	WWTP	sample	Protocol Z			Protocol NS1		
			+ replicates / 5	mΔCq	mean GC/L of RNA (E gene)	+ replicates / 5	mΔCq	mean GC/L of RNA (E gene)
1	1	S1	5/5	3.4 ± 0.7	2.5 × 10^5^ ± 6.1 × 10^4^	5/5	2.5 ± 0.3	3.3 × 10^5^ ± 2.0 × 10^5^
		S2	5/5	3.1 ± 0.7	2.0 × 10^5^ ± 3.7 × 10^4^	5/5	3.7 ± 1.6	1.6 × 10^5^ ± 1.1 × 10^5^
		S3	5/5	3.3 ± 0.4	7.1 × 10^4^ ± 4.0 × 10^4^	5/5	2.2 ± 1.0	9.0 × 10^4^ ± 6.0 × 10^4^
	2	S4	5/5	3.1 ± 0.2	5.6 × 10^4^ ± 2.0 × 10^4^	5/5	3.1 ± 0.6	1.5 × 10^5^ ± 7.0 × 10^4^
		S5	5/5	2.9 ± 0.2	1.7 × 10^5^ ± 5.3 × 10^4^	5/5	1.5 ± 1.4	3.0 × 10^5^ ± 4.0 × 10^4^
		S6	4/5	3.2 ± 0.7	2.0 × 10^4^ ± 1.3 × 10^4^	5/5	2.8 ± 0.2	3.0 × 10^4^ ± 1.0 × 10^4^
	3	S7	4/5	3.2 ± 0.2	3.9 × 10^4^ ± 3.2 × 10^4^	5/5	2.4 ± 1.7	2.8 × 10^5^ ± 1.5 × 10^5^
		S8	4/5	2.9 ± 0.5	1.3 × 10^5^ ± 3.5 × 10^4^	5/5	2.6 ± 0.7	1.7 × 10^5^ ± 1.1 × 10^5^
		S9	3/5	3.8 ± 0.8	8.5 × 10^3^ ± 5.5 × 10^3^	4/5	2.0 ± 0.7	3.0 × 10^4^ ± 1.0 × 10^4^
			40/45			44/45		


			Protocol Z			Protocol NS2		
			+ replicates / 5	mΔCq	mean GC/L of RNA (E gene)	+ replicates / 5	mΔCq	mean GC/L of RNA (E gene)
2	4	S10	5/5	2.8 ± 0.4	3.7 × 10^5^ ± 1.9 × 10^5^	5/5	3.7 ± 0.2	7.6 × 10^5^ ± 4.7 × 10^5^
		S11	5/5	3.5 ± 0.3	3.7 × 10^5^ ± 1.0 × 10^5^	5/5	3.5 ± 0.2	9.4 × 10^5^ ± 8.6 × 10^5^
		S12	5/5	3.3 ± 0.3	2.2 × 10^5^ ± 9.0 × 10^4^	5/5	3.7 ± 0.2	5.0 × 10^5^ ± 2.2 × 10^5^
	5	S13	5/5	3.6 ± 0.6	2.0 × 10^4^ ± 2.0 × 10^4^	5/5	3.7 ± 0.2	2.0 × 10^4^ ± 1.0 × 10^4^
		S14	5/5	3.7 ± 0.4	1.0 × 10^4^ ± 0	5/5	3.6 ± 0.2	2.0 × 10^4^ ± 1.0 × 10^4^
		S15	5/5	3.5 ± 0.5	7.0 × 10^4^ ± 3.0 × 10^4^	5/5	3.6 ± 0.2	1.0 × 10^5^ ± 3.0 × 10^4^
	6	S16	5/5	3.8 ± 0.4	2.4 × 10^5^ ± 3.5 × 10^5^	5/5	3.9 ± 0.1	1.9 × 10^5^ ± 1.2 × 10^5^
		S17	5/5	3.3 ± 0.3	1.1 × 10^5^ ± 2.0 × 10^4^	5/5	3.5 ± 0.1	1.1 × 10^5^ ± 1.0 × 10^4^
		S18	5/5	2.8 ± 0.5	1.4 × 10^5^ ± 2.0 × 10^4^	5/5	3.6 ± 0.2	1.4 × 10^5^ ± 3.0 × 10^4^
			45/45			45/45		


			Protocol Z			Protocol SB		
			+ replicates / 5	mΔCq	mean GC/L of RNA (E gene)	+ replicates / 5	mΔCq	mean GC/L of RNA (E gene)
3	7	S19	5/5	3.5 ± 0.3	2.0 × 10^4^ ± 0	5/5	2.2 ± 0.2	3.0 × 10^4^ ± 1.0 × 10^4^
		S20	4/5	3.6 ± 0.3	1.7 × 10^5^ ± 4.0 × 10^4^	5/5	1.4 ± 0.6	6.0 × 10^4^ ± 2.0 × 10^4^
		S21	5/5	3.3 ± 0.4	7.0 × 10^4^ ± 1.0 × 10^4^	5/5	2.8 ± 0.1	2.0 × 10^4^±1.0 × 10^4^
	8	S22	4/5	3.5 ± 0.5	1.0 × 10^5^ ± 2.0 × 10^4^	5/5	2.8 ± 0.4	5.0 × 10^4^±1.0 × 10^4^
		S23	5/5	3.8 ± 0.5	3.0 × 10^4^ ± 1.0 × 10^4^	5/5	1.8 ± 0.2	1.0 × 10^4^±1.0 × 10^4^
		S24	5/5	3.6 ± 0.3	4.0 × 10^4^ ± 2.0 × 10^4^	5/5	2.4 ± 0.6	4.0 × 104 ± 1.0 × 104
	9	S25	0/5	ND	ND	5/5	2.8 ± 0.5	8.0 × 10^4^ ± 1.0 × 10^4^
		S26	5/5	4.1 ± 0.9	3.0 × 10^4^ ± 1.0 × 10^4^	5/5	2.3 ± 0.5	1.0 × 10^4^ ± 1.0 × 10^4^
		S27	5/5	3.6 ± 0.6	3.0 × 10^4^ ± 1.0 × 10^4^	5/5	2.5 ± 0.5	4.0 × 10^4^ ± 2.0 × 10^4^
			38/45			45/45		

Z, NS1, NS2, and SB refer to extraction protocols described in Fig. 1. ND (Not Detected).

mΔCq: mean of the ΔCq (difference between Cq value of 1/10 diluted extract and Cq value of undiluted extract) for the 5 replicates of one sample.

## Conclusion

This study compared a WW-designed RNA extraction protocol (Z) with three non-WW-designed protocols across three laboratories, each using locally collected paired WW samples. The NS2—described in detail above— involves additional costs (twice the cost of the Z protocol) and requires careful chemical handling. However, it was the only one that effectively concentrated the SARS-CoV-2 genome while also removing RT-qPCR inhibitors more efficiently than the Z protocol (results are shown above in the protocol validation). Additionally, when the objective is to process large volumes of wastewater to enhance sensitivity—as in the related study [1]—inhibition becomes a critical challenge, as inhibitors are often co-concentrated along with the target material. In this context, the phenol-chloroform purification step used in the NS2 protocol can be particularly important. This is due to the limited availability of effective alternative methods for inhibitor removal under such conditions.

## Limitations

This study was conducted across three laboratories using WW samples collected locally, reflecting real-world conditions and operational variability. While this approach enhances the practical relevance of the findings, it may also introduce some variability between samples. Additionally, only one SARS-CoV-2 genomic target was assessed; incorporating additional genomic regions in future analyses would allow for a more comprehensive evaluation of protocol performance across diverse WW matrices. Although the adaptability of the protocols to larger sample volumes was not experimentally tested in this study, it was thoroughly assessed in the related original study [1].

## CRediT authorship contribution statement

**Ahlam Chaqroun:** Writing – original draft, Visualization, Methodology, Investigation, Formal analysis. **Ghina El Soufi:** Writing – review & editing, Investigation, Formal analysis. **Zuzana Gerber:** Writing – review & editing, Visualization, Investigation, Formal analysis. **Julie Loutreul:** Investigation, Formal analysis. **Nicolas Cluzel:** Writing – review & editing, Visualization, Formal analysis. **Julie Challant:** Methodology, Writing – review & editing. **Damien Delafoy:** Formal analysis. **Florian Sandron:** Formal analysis. **Léo Di Jorio:** Investigation. **Stéphanie Raffestin:** Resources. **Vincent Maréchal:** Writing – review & editing, Supervision, Funding acquisition. **Christophe Gantzer:** Writing – review & editing. **Robert Olaso:** Supervision. **Jean-François Deleuze:** Supervision, Funding acquisition, Conceptualization. **Olivier Rohr:** Writing – review & editing, Supervision, Resources, Funding acquisition, Conceptualization. **Nicolas Boudaud:** Writing – review & editing, Validation, Supervision, Resources, Project administration, Methodology, Funding acquisition, Conceptualization. **Clémentine Wallet:** Writing – review & editing, Validation, Supervision, Resources, Project administration, Methodology, Conceptualization. **Isabelle Bertrand:** Writing – review & editing, Visualization, Validation, Supervision, Resources, Project administration, Methodology, Funding acquisition, Conceptualization.

## Declaration of competing interest

The authors declare that they have no known competing financial interests or personal relationships that could have appeared to influence the work reported in this paper.

## Data Availability

Data will be made available on request.
